# Prevalence of Gynaecomastia in Male Pakistani Population

**Published:** 2017-01

**Authors:** Muhammad Ahmad

**Affiliations:** Plastic, Reconstructive, Hand and Hair Restorative Surgeon, La Chirurgie, Islamabad Cosmetic Surgery Centre, Islamabad, Pakistan

**Keywords:** Gynaecomastia, Men, Male, Prevalence, Pakistan

**DEAR EDITOR**

Gynaecomastia has an overall incidence of 32 to 40%.^1^ The highest prevalaence is found in old age and up to 65% in men.^2^ The breast tissue growth is dependent on the balance between the oestrogens and androgens; thus any physiological or pathological factor that can interfere with this balance, can result in gynaecomastia.^2^ Physiologically, it can be neonatal, pubertal or senile. Pathologically, it can occur in various diseases, e.g., hepatic disease, renal diseases, thyrotoxicosis,^3^ certain cancers like Sertoli cell tumour, bronchial carcinoma, lymphoma, gastric carcinoma,^3^ with the use of certain drugs, e.g., cimitidine, spironolactone, oestrogens, etc.^3^ Various pharmacological drugs have been tried but surgery remains the mainstay of the treatment.^4^

Gynaecomastia has a negative impact on self-confidence and body image.^5^ It should be distinguished from pesudogynaecomastia which is common in obese men and consists of lipomatesia without glandular proliferation.^5^ Treatment must be individualized while surgery is planned depending on the grade and histopathology of gynaecomastia.^6^ Liposuction is beneficial but patients presenting with larger enlargement, need skin retailoring surgery.^7^ The main objective of the following study was to present the demographic details and the operative measures for the patients presenting with gynaecomastia. 

All patients with gynaecomastia from January 2006 to December 2013 were included. A detailed history was taken with regard to the age of onset, duration of the enlargement, recent changes in the size, and any discharge. Any history of pain, alcohol intake or any other drugs was also noted. The patients presenting with lesion other than benign enlargement (cancer or abscess) were excluded from the study. All the patients were thoroughly examined. All the patients underwent routine laboratory tests and ultrasound check-up. Patients presenting with mild to moderate enlargement underwent Power-assisted liposuction (PAL). Patients presenting with severe enlargement and ptosis underwent excision as well as liposuction using nipple-areola complex (NAC) repositioning and excision of the excess skin. The patients were followed-up for six months and any complication was noted.

Totally, 77 patients were enrolled. The mean age of the patients was 31.5 years ranging from 18 to 46 years. All the patients had bilateral enlargement. Majority of the patients (48.1%) belonged to 25–35 years age group (<25 y: 18.2%; 36-45 y: 31.2% and >46 years: 2.5%). In majority of the patients (77.3%), the cause was not known, only 4.5% had history of drug intake. Type I small enlargement of breast tissue was noted in 27.9% of patients and 67.6%, 1.5% and 2.9% were visible in types IIA, IIB and III, respectively. Liposuction with surgical excision (57.4%), liposuction only (41.2%) and surgery only (1.5%) were the mainstay treatments. 

Drains were placed in only three cases with type III enlargement. A few complications were noted such as skin ecchymosis (13.2%) with seroma , scar revision and disc deformity (1.5%) in only one patient. No case of infection was seen. One patient needed revision surgery. Respectively, the findings of a 24 and a 34 years old patient with gynaecomastia for 5 and 10 years who underwent liposuction alone and liposuction with surgical excision were satisfied with treatment modality.

The incidence of gynaecomastia varies from 30% to 65%.^8^ There is no available data for the prevalence of this disease in Pakistan, however, a few studies have been reported.^9^ In our setup, relatively fewer patients reported to the surgeons for this disease, probably due to shame or guilt. It is a source of embarrassment, surprise and concern to the young as well as the elder men. The usual reason for the presentation is that young men do not like having breasts and older men are worried about the possibility of the cancer.^9^ The average age of presentation in our study was 31.6 year which is similar to the observation noted by other studies.^10^

Whereas 55% and 87% patients were presented with the age group less than 30 years which are in contrast to the present study where the patients (<30 years) were only 42.9%.^9^ As with other studies carried out, 77% of the patients had no specific reason for the gynaecomastia.^11^ No medical treatment was given to any patient in the present study, whereas 12% and 20% patients received medical treatment in other studies.^9^ The gynaecomastia may need volume reduction surgery with or without skin retailoring surgery.^12^ These surgical options include semi-circular intra-areolar incision, superior peri-areolar incision with skin excision, trans-axillary approach, suction-assisted lipectomy, ultrasound-assisted liposuction etc.^13^ The choice of the surgical option basically depends on the severity of the tissue development, skin excess and the surgeon’s experience and comfort with the various techniques.

the periareolar subcutaneous mastectomy was previously performed for the removal of the extra breast tissue.^9^ The main drawback with this type of the surgery is the possible complication of the disc-deformity which can be avoided by leaving an ample amount of the tissue at NAC or combining liposuction. The other potential complication could be exact placement of the periareolar scar, i.e., at the junction of pigmented areolar skin with the on-pigmented skin. The improper placement can result in abnormal scarring. 

Only the first two patients in the current series underwent the peri-areolar mastectomy alone. Majority of the patients (70%) in the present study underwent power-assisted liposuction combined with surgical excision. In the study by Mageed, 77.8% of the patients underwent liposuction which is in contrast to the present study where only 27% of the patients underwent liposuction alone.^14^ In a small series of 12 patients (grade IIb and III), a combined approach was undertaken.^15^ A complication rate of 25% was observed which is higher than the current study. Another study by Mishra *et al.* used trans-nipple removal, avoiding the peri-areolar scar.^16^ In the present study, only PAL (Power-Assisted Liposuction) was performed in all the patients whereas in the study by Wong *et al. *in which UAL (ultrasound-assisted liposuction) was found to be more effective than conventional liposuction.^10^

Only 9% of the patients undergoing liposuction developed skin ecchymosis which was relieved spontaneously after 4 weeks. Only one case of seroma was noted undergoing skin excision. This patient presented with very severe gynaecomastia. No case of infection or haematoma was found. Disc-deformity was noted the first case of the current series which was corrected by liposuction after 8 months postoperatively. One case of abnormal scarring was noticed. This was the same patient developing seroma which resulted in higher morbidity. Gynaecomastia can be treated satisfactorily with the liposuction becoming the treatment of the choice. However, the surgical excision of the severe deformity can also be combined to achieve the aesthetically pleasing results. 

## CONFLICT OF INTEREST

The authors declare no conflict of interest.
